# The Modulation of Phase II Drug-Metabolizing Enzymes in Proliferating and Differentiated CaCo-2 Cells by Hop-Derived Prenylflavonoids

**DOI:** 10.3390/nu12072138

**Published:** 2020-07-18

**Authors:** Kateřina Lněničková, Michaela Šadibolová, Petra Matoušková, Barbora Szotáková, Lenka Skálová, Iva Boušová

**Affiliations:** 1 Faculty of Medicine and Dentistry, Palacký University, Hněvotínská 3, 77515 Olomouc, Czech Republic; katerina.lnenickova@upol.cz; 2 Faculty of Pharmacy in Hradec Králové, Charles University, Heyrovského 1203, 50005 Hradec Králové, Czech Republic; sadibolom@faf.cuni.cz (M.Š.); matousp7@faf.cuni.cz (P.M.); szotakova@faf.cuni.cz (B.S.); skaloval@faf.cuni.cz (L.S.)

**Keywords:** prenylflavonoid, xanthohumol, enzymatic activity, mRNA expression, glutathione S-transferase, catechol-O-methyltransferase, sulfotransferase, UDP-glucuronosyl transferase, CaCo-2 cells

## Abstract

Prenylflavonoids in the human organism exhibit various health-beneficial activities, although they may interfere with drugs via the modulation of the expression and/or activity of drug-metabolizing enzymes. As intestinal cells are exposed to the highest concentrations of prenylflavonoids, we decided to study the cytotoxicity and modulatory effects of the four main hop-derived prenylflavonoids on the activities and mRNA expression of the main drug-conjugating enzymes in human CaCo-2 cells. Proliferating CaCo-2 cells were used for these purposes as a model of colorectal cancer cells, and differentiated CaCo-2 cells were used as an enterocyte-like model. All the tested prenylflavonoids inhibited the CaCo-2 cells proliferation, with xanthohumol proving the most effective (IC_50_ 8.5 µM). The prenylflavonoids modulated the activities and expressions of the studied enzymes to a greater extent in the differentiated, as opposed to the proliferating, CaCo-2 cells. In the differentiated cells, all the prenylflavonoids caused a marked increase in glutathione S-transferase and catechol-O-methyltransferase activities, while the activity of sulfotransferase was significantly inhibited. Moreover, the prenylflavonoids upregulated the mRNA expression of uridine diphosphate (UDP)-glucuronosyl transferase 1A6 and downregulated that of glutathione S-transferase 1A1/2.

## 1. Introduction

Dietary factors may play a crucial role in the prevention and progression of various lifestyle diseases, such as diabetes mellitus, cancer, obesity and cardiovascular diseases. According to epidemiological studies, a higher consumption of fruits, vegetables, nuts, legumes and whole grains, and a lower consumption of sugar-sweetened food and beverages, red/processed meat and refined grains, are associated with a lower risk of type II diabetes mellitus and cancer [1]. An increased dietary intake of antioxidants such as polyphenols, vitamins (C, E) and carotenoids may be an important factor in the prevention of lifestyle diseases. In particular, flavonoids, which represent approximately two-thirds of all dietary polyphenols, possess multiple biological and pharmacological activities (e.g., antioxidant, anti-inflammatory, anti-cancer activities) [2].

Prenylflavonoids, one sub-class of flavonoids with a distribution restricted to several plant families (e.g., Moraceae and Fabaceae), have attracted increasing attention due to their potential benefits for human health. Prenylflavonoids possess a basic diphenyl propane skeleton with a prenyl (C5 isoprene) group(s) attached to different positions of the aromatic ring. One rich source of prenylflavonoids are hops, i.e., the strobili of *Humulus lupulus L.* (Cannabaceae), which are used in the brewing of beer and as a component of estrogenic dietary supplements for menopausal women [3]. Hops contain mainly prenylated chalcone xanthohumol (XH) and prenylated flavanones, including isoxanthohumol (IXH), 6-prenylnarigenin (6-PN), and 8-prenylnarigenin (8-PN) [4]. Prenylation enhances the biological activities of prenylflavonoids and tissue bioaccumulation compared to their unprenylated counterparts [5,6]. The 8-prenylquercetin revealed two-fold stronger anti-inflammatory properties in vitro as well as in vivo than quercetin did [7]. The prenylated flavonoids, XH, IXH, 6-PN and 8-PN, showed stronger cytotoxic effects in human colorectal cancer cell lines than did the parent compound naringenin [8]. Multiple biological targets of prenylflavonoids, such as aromatase, the aryl hydrocarbon receptor, nuclear factor NF-κB and the Kelch-like ECH-associated protein 1 (Keap1)/nuclear factor erythroid-2-related factor 2 (Nrf2)/antioxidant responsive element (ARE) pathway, have been identified and reviewed in [4].

Like other xenobiotics, prenylflavonoids are metabolized by drug-metabolizing enzymes (DMEs) during absorption and circulation. The intestinal microflora plays also an important role in the biotransformation of prenylflavonoids. Following a XH oral intake, IXH, 6-PN and 8-PN have been identified as its metabolites in the human gastrointestinal tract [9,10]. However, the most abundant metabolites of all the prenylflavonoids identified in the plasma and urine of human volunteers were glucuronides [9]. Prenylflavonoids have been reported to influence the activity and/or expression of various DMEs. In terms of phase I DMEs, the inhibitory activity of XH and its related prenylflavonoids towards human carbonyl reductase 1 [11], aldo–keto reductase 1B1 and 1B10 [12], cytochrome P450 1A1/2, 1B1 [13], 2C8, 2C9, and 2C19 [3] have been described in vitro. Regarding phase II DMEs, the gene expression of glutathione S-transferase A1 (GSTA1) has been shown to be induced by prenylflavonoids via the Keap1–Nrf2–ARE pathway [14,15]. In rats, an oral administration of XH led to a decreased mRNA expression of sulfotransferase 1A1 (SULT) and UDP-glucuronosyl transferase 1A1 (UGT1A1) in the liver [16].

Therefore, it can assumed that the consumption of prenylflavonoids (in food and dietary supplements) may modulate the activity and expression of DMEs with potential pharmacological and/or toxicological consequences [3]. Due to a low absorption rate of the ingested prenylflavonoids into the circulation, intestinal cells are exposed to the highest concentrations of these compounds and the intestinal DMEs could be the most affected. However, the effect of these compounds on the enzymatic activity and expression of intestinal DMEs has not yet been studied. Moreover, the effect of prenylflavonoids on the DMEs could differ in proliferating and differentiated intestinal cells. Knowledge of these differences could facilitate evaluations of the benefits and/or risks of prenylflavonoid consumption, particularly during cancer prevention and treatment.

In the present in vitro study, the cytotoxicity and modulatory activity of the hop-derived prenylflavonoids xanthohumol (XH), isoxanthohumol (IXH), 6-prenylnaringenin (6-PN) and 8-prenylnaringenin (8-PN) on the activities and mRNA expression of the main phase II DMEs glutathione S-transferases, UDP-glucuronosyl transferases, sulfotransferases, and catechol-O-methyltransferases (COMT) were studied and compared in proliferating and differentiated CaCo-2 cells. These cancerous cells undergo spontaneous differentiation into enterocyte-like cells after reaching confluence. The morphological and functional characteristics of differentiated CaCo-2 cells are similar to those of mature enterocytes, including drug metabolism, transport and barrier functions [17]. A comparison of the effects of prenylflavonoids on the activity and mRNA expression of DMEs in the cancerous and non-cancerous cells has not been performed yet.

## 2. Materials and Methods

### 2.1. Chemicals and Reagents

Minimum essential medium (MEM) non-essential amino acid solution as well as Eagle’s MEM (EMEM), L-glutamine solution, xanthohumol (XH), isoxanthohumol (IXH), 6-prenylnaringenin (6-PN), 8-prenylnaringenin (8-PN), L-(−)-norepinephrine, S-(5′-Adenosyl)-L-methionine (SAM), L-glutathione (GSH), 1-chloro-2,4-dinitrobenzene (CDNB), 3′-phosphoadenosine 5′-phosphate (PAP), 2-naphthol and menadione were obtained from Merck (Kenilworth, NJ, USA). Fetal bovine serum and streptomycin sulfate were purchased from Invitrogen (Carlsbad, CA, USA). All other chemicals of HPLC or analytical grade were obtained from Sigma-Aldrich (Prague, Czech Republic). All prenylflavonoids were dissolved in dimethyl sulfoxide (DMSO) and their stock solutions were stored at 4 °C in the dark.

### 2.2. Cultivation of the CaCo-2 Cell Line

The human epithelial colorectal adenocarcinoma CaCo-2 cell line was purchased from ATCC (supplier for Czech Republic: LGC Standards, Kielpin, Poland). The cells were cultured in EMEM supplemented with 10% (v/v) heat-inactivated fetal bovine serum, 1% (v/v) non-essential amino acids, 1% (v/v) glutamine, and 0.5% penicillin/streptomycin. The cells were grown in Petri dishes for 3 days and 21 days before treatment in a humidified atmosphere containing 5% CO_2_ at 37 °C to obtain proliferating and differentiated cells, respectively. The medium was changed twice a week.

### 2.3. Treatment of CaCo-2 Cells with Prenylated Flavonoids

Proliferating and differentiated cells were treated with a single dose of xanthohumol (XH), isoxanthohumol (IXH), 6-prenylnaringenin (6PN) and 8-prenylnaringenin (8PN) in a final concentration of 1 µM. After 24/72 h of incubation, the cells were rinsed with phosphate-buffered saline (PBS) and they were harvested into a 0.1 M sodium phosphate buffer (pH 7.4) or TriReagent (a mixture of guanidine thiocyanate and phenol in a monophase solution; Sigma-Aldrich, Prague, Czech Republic). Cell suspensions were stored at −80 °C, until further processing (subcellular fractions preparation/mRNA isolation).

The viability of the CaCo-2 cells after a 24 and 72 h of exposure to the tested compounds was assessed using the neutral red uptake (NRU) assay as described in our previous report [8].

### 2.4. Preparation of Subcellular Fractions

Microsomal and cytosolic fractions were obtained from suspensions of the control (0.01% of DMSO) and the influenced CaCo-2 cells. The cells were homogenized using sonication with the homogenizer Sonopuls (Bandelin, Germany). Differential centrifugation was used to isolate the subcellular fractions as described previously [18]. The resultant subcellular fractions were stored at −80 °C.

### 2.5. Enzymatic Activity Assessment

The enzymatic activities of the selected conjugating enzymes were assayed in cytosolic or mitochondrial fractions of proliferating, as well as differentiated, CaCo-2 cells. Each enzymatic activity assay was repeated three times with 5–6 parallel measurements in all repetitions. The determination of the GST, SULT and UGT activity was based on the spectrophotometric detection of the formed product or the decreasing level of substrate/cofactor using the Infinite M200PRO microplate reader UV-VIS spectrophotometer (Tecan, Männedorf, Switzerland).

The cytosolic glutathione S-transferase (GST) activity was assayed according to spectrophotometric method of Habig [19] with some modifications [20]. Briefly, this kinetic method is based on the spectrophotometric detection of the formed 2,4-dinitrochlorobenzene-glutathione conjugate, the product of a GST catalyzed reaction. The absorbance was measured at 340 nm by the Tecan Infinite M200PRO.

The method used in this study for the SULT activity assay was described by Frame et al. [21] with some modifications [20]. The assay depends on the formation of 4-nitrophenol and 3′-phosphoadenosine-5′-phosphosulfate from 4-nitrophenolsulphate and PAP. The increasing level of 4-nitrophenol, which was measured at a single time point, correlates with an absorbance growth at 405 nm, as measured by the Tecan Infinite M200PRO.

The determination of the UDP-glucuronosyltransferase (UGT) activity in the microsomal fractions (2 mg of protein/mL) was carried out according to the method of Letelier et al. [22] with some modifications [20]. The principle of this method is based on the single time point measurement of the absorbance of the remaining 4-nitrophenol at 405 nm.

The cytosolic catechol-O-methyltransferase (COMT) activity was measured according the method of Aoyama et al. [23] with some modifications [18]. This activity assay is based on the HPLC detection of normetanephrine, which is formed from norepinephrine by COMT.

The catalytic activity of each enzyme was normalized to mg of protein in cytosolic/microsomal fractions. The protein concentrations in the subcellular fractions were assayed using the bicinchoninic acid assay according to the Sigma-Aldrich protocol.

### 2.6. Determination of mRNA Expression

The total RNA was isolated using TriReagent according to the manufacturer’s instructions (Biotech, Prague, Czech Republic). The concentration and purity of RNA were determined spectrophotometrically using a NanoDrop ND-1000 UV-Vis Spectrophotometer (Thermo Scientific, Waltham, MA, USA). The synthesis of first strand cDNA proceeded from 1 µg of total RNA and 1 µL of 50 µM random hexamers (Generi Biotech, Hradec Kralove, Czech Republic) using ProtoScript II reverse transcriptase (New England Biolabs, Ipswich, MA, USA). The qPCR Core kit for SYBR Green I (Eurogentec, Seraing, Belgium) was used for qPCR analyses, which were performed using a QuantStudio 6 Flex (Applied Biosystems, Foster City, CA, USA). The primer sequences are listed in Table 1. The relative expression levels of the target genes were calculated using the 2^−ΔΔCt^ method [24]. The normalized expression level was expressed using a geometric mean of reference genes (beta-2 microglobulin, B2M; glyceraldehyde 3-phosphate dehydrogenase, GAPDH). The obtained data were expressed as the fold change relative to the control.

### 2.7. Statistical Analysis

The presented data were obtained from three independent measurements. The Microsoft Excel and GraphPad Prism 7.03 (GraphPad Software, San Diego, CA, USA) were used to process all data. A one-way ANOVA with Dunnett’s post-hoc test was used for the statistical evaluation of the differences between the treated groups and controls. The differences were considered as statistically significant at *p* < 0.05.

## 3. Results

This study was focused on the evaluation of the effect of four commonly occurring prenylflavonoids, namely xanthohumol (XH), isoxanthohumol (IXH), 6-prenylnaringenin (6-PN) and 8-prenylnaringenin (8-PN), on the activity and mRNA expression of DMEs in proliferating, as well as differentiated, CaCo-2 cells. The enzymes selected for this study, i.e., glutathione S-transferase (GST), sulfotransferase (SULT), catechol-O-methyltransferase (COMT) and UDP-glucuronosyl transferase (UGT), represent the main phase II DMEs [25].

### 3.1. Cytotoxicity of Prenylflavonoids

**Figure 1:**
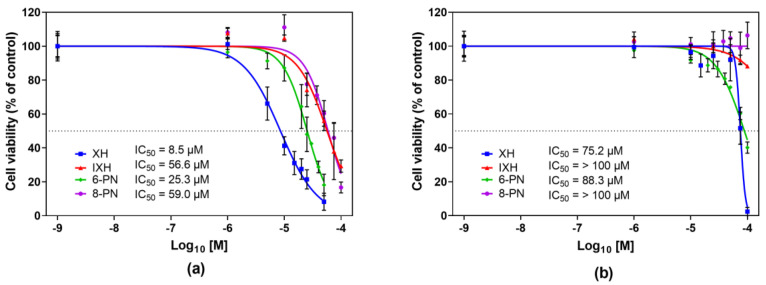
The effect of xanthohumol, isoxanthohumol, 6-prenylnaringenin and 8-prenylnaringenin on the viability of proliferating (**a**) and differentiated (**b**) CaCo-2 cells after a 24 h treatment. Cell viability was assayed using the neutral red uptake assay. Data presented as a percentage of the respective control (=100%) represent the mean ± S.D. of 3–4 independent experiments. IC_50_ values were calculated using GraphPad Prism 8 software as an average of 3–4 independent experiments.

At the outset, the cytotoxicity of individual prenylflavonoids against proliferating, as well as differentiated, CaCo-2 cells was evaluated. As the effect of prenylflavonoids on the enzymatic activity and mRNA expression was monitored at two time points (24 h and 72 h), the cytotoxicity of these compounds was studied at both intervals as well. Since the cytotoxicity of prenylflavonoids after a 72 h incubation was published in our previous report [8], only the results obtained after 24 h incubation are presented in Figure 1.

The proliferating and differentiated cells were exposed to individual prenylflavonoids within the concentration range of 0–100 µM, and cell viability was measured using the neutral red uptake (NRU) assay. All the studied prenylflavonoids exerted a higher cytotoxicity towards proliferating than differentiated CaCo-2 cells, with XH being the most effective in both cell lines and at both incubation times. In the proliferating cells, the concentration-dependent decrease in the cell viability was observed for all the studied prenylflavonoids at both monitored time points, while IXH and 8-PN showed only a feeble effect on the viability of the differentiated cells (Figure 1). The half maximal inhibitory concentration (IC_50_) values are shown in Figure 1.

The IC_50_ values obtained after 72 h of incubation were lower for all prenylflavonoids compared to those after 24 h. In accordance with the data after 24 h, the cytotoxic effect of all the prenylflavonoids was more pronounced in proliferating cells than in differentiated cells, and XH was the compound with the strongest antiproliferative effect. The IC_50_ ranged from 6.2 µM to 48.9 µM in proliferating cells and from 39.3 µM to >100 µM in differentiated cells [8].

### 3.2. The Enzymatic Activity and mRNA Expression of DMEs in Proliferating and Differentiated CaCo-2 Cells

The enzymatic activities of GST, SULT, COMT and UGT were assessed using spectrophotometric methods in the proliferating, as well as differentiated, cells. However, the enzymatic activity of UGT was not detected in either cell type. In the course of differentiation, the enzymatic activities of all the DMEs significantly increased as compared to the proliferating cells (see Table 2).

The mRNA expression of the studied phase II DMEs was measured using RT-qPCR. PCR primers were designed to detect GSTA1/2, GSTP1, SULT1A1/2, COMT and UGT1A6 isoforms. In the proliferating cells, the expression of UGT1A6 mRNA was not detected. In the differentiated cells, the mRNA expression of GSTA1/2, GSTP1, SULT1A1/2 and COMT was significantly higher than in the proliferating cells (see Table 2).

### 3.3. Modulation of DMEs Expression by Prenylflavonoids

Subsequently, the effect of prenylflavonoids on the mRNA expression of the selected DMEs was studied both in the proliferating cells and in the differentiated cells. The CaCo-2 cells were cultivated in the presence of individual prenylflavonoids and DMSO (control) for 24 and 72 h. Based on the results of the cell viability, a 1 µM concentration of prenylflavonoids (non-toxic in both cell lines) was used. PCR primers were designed to detect GSTA1/2, GSTP1, SULT1A1/2, COMT and UGTA6 isoforms.

**Figure 2:**
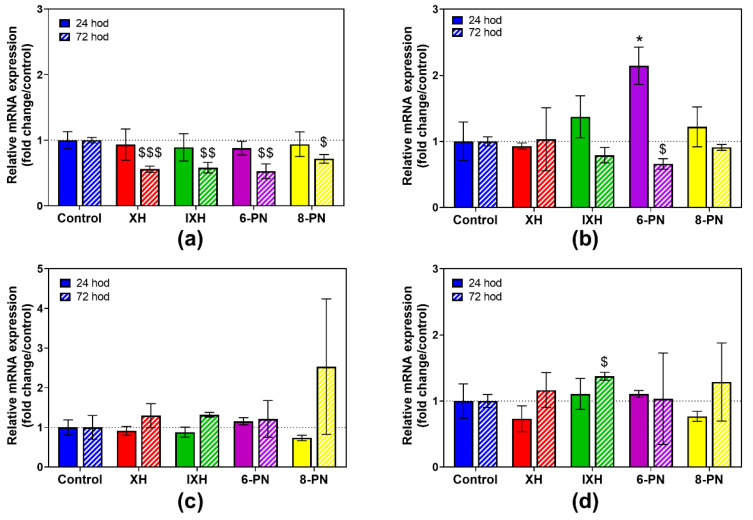
The effect of prenylflavonoids (1 µM) on the normalized mRNA expression of GSTA1/2 (**a**), GSTP1 (**b**), SULT1A1 (**c**) and COMT (**d**) in proliferating CaCo-2 cells after 24 h and 72 h (*n* = 3). The normalized expression level was calculated using the 2^−ΔΔCt^ method with the geometric mean of glyceraldehyde 3-phosphate dehydrogenase (GAPDH) and beta-2 microglobulin (B2M) as a reference gene. Results are presented as the mean ± SD of three independent experiments. Statistical analyses were performed using a one-way ANOVA with Dunnett’s test: *p* < 0.05 (* or §), *p* < 0.01 (§§), *p* < 0.001 (§§§); * different from control after 24 h, $ different from control after 72 h.

The expression of UGT1A6 was not detected in the proliferating cells. After 24 h of incubation, the influence of the prenylflavonoids on the mRNA expression of the studied DMEs was rather feeble, except for 6-PN, which induced GSTP1 mRNA 2.1 times (Figure 2b). The GSTA expression achieved a six-fold upregulation in the controls after 72 h of incubation (Figure S1), and all the prenylflavonoids significantly reduced this increase by 28.3% to 47.2% (Figure 2a).

**Figure 3:**
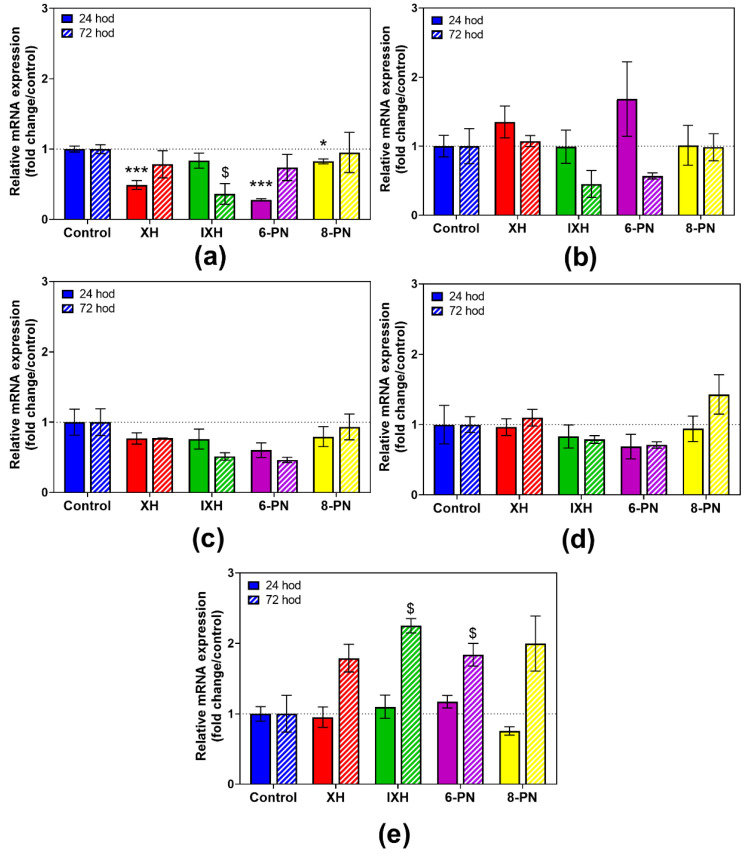
The effect of prenylflavonoids (1 µM) on the normalized mRNA expression of GSTA1/2 (**a**), GSTP1 (**b**), SULT1A1 (**c**), COMT (**d**) and UGT1A6 (**e**) in differentiated CaCo-2 cells after 24 h and 72 h (*n* = 3). The normalized expression level was calculated using the 2^−ΔΔCt^ method with the geometric mean of GAPDH and B2M as a reference gene. Results are presented as the mean ± SD of 3 independent experiments. Statistical analyses were performed using a one-way ANOVA with Dunnett’s test: *p* < 0.05 (* or §), *p* < 0.001 (***); * different from control after 24 h, $ different from control after 72 h.

In the differentiated CaCo-2 cells, XH, 6-PN and 8-PN caused a significant decrease in GSTA1/2 mRNA after 24 h of incubation (Figure 3a). Particularly 6-PN showed a strong inhibitory effect on GSTA1/2 expression, as this compound reduced mRNA expression by 72.2%. Presumed increase in GSTA1/2 mRNA in the samples containing XH and 6-PN after 72 h compared to those after 24 h is probably caused by the upregulation of total GSTA1/2 mRNA. In control samples, 1.7-fold increase in GSTA1/2 mRNA was observed after 72 h (Figure S1a). In the case of UGT1A6, all the prenylflavonoids caused a marked increase in its respective mRNA expression by 78.9% to 125.0% compared to the control after 72 h (Figure 3e).

### 3.4. Effect of Prenylflavonoids on DMEs Activities

Based on the results of the cell viability assay, the effect of prenylflavonoids on the activity of selected phase II DMEs was studied at 1 µM concentration, which was shown as non-toxic for all the prenylflavonoids in both cell lines.

**Figure 4:**
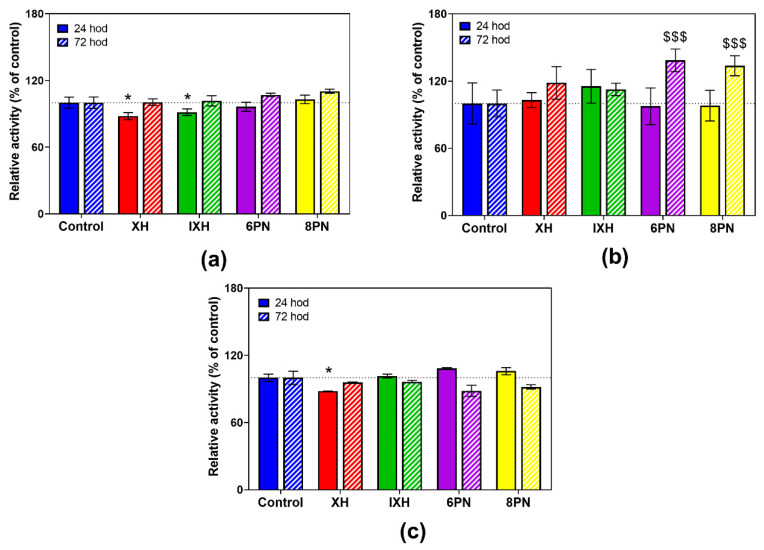
The effect of prenylflavonoids on the enzymatic activity of glutathione S-transferase (**a**), sulfotransferase (**b**) and catechol-O-methyltransferase (**c**) in the proliferating CaCo-2 cells after a 24 h and 72 h treatment with 1 µM of prenylflavonoid. The enzymatic activities were assayed using colorimetric assays. Data presented as percentage of the respective control (=100%) represent the mean ± S.D. of 3–4 independent experiments. Statistical analyses were performed using a one-way ANOVA with Dunnett’s test: *p* < 0.05 (*), *p* < 0.001 (§§§); * different from control after 24 h, $ different from control after 72 h.

In the proliferating cells, the tested prenylflavonoids only mildly reduced the GST and COMT enzymatic activity. Xanthohumol decreased the activity of those enzymes after 24 h of incubation by 12.0% and 11.9%, respectively. After 72 h, no inhibition was observed, probably due to the increase in the GST expression as a 5.9-fold upregulation in GSTA1/2 expression was observed in control samples after 72 h (Figure S1). Conversely, 6-PN and 8-PN increased the activity of SULT by 36.8% and 33.7% after 72 h of incubation, respectively (Figure 4).

**Figure 5:**
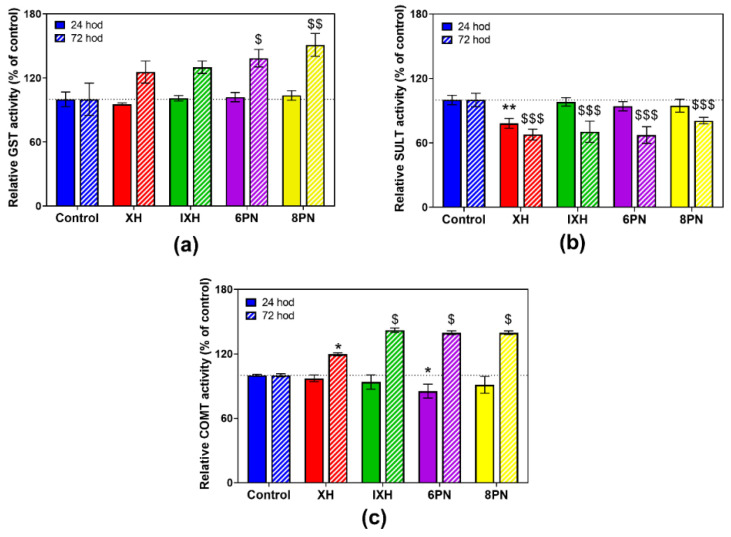
The effect of prenylflavonoids on the enzymatic activity of glutathione S-transferase (**a**), sulfotransferase (**b**) and catechol-O-methyltransferase (**c**) in the differentiated CaCo-2 cells after 24 h and 72 h treatment with 1 µM of prenylflavonoid. The enzymatic activities were assayed using the colorimetric assays. Data presented as percentage of respective control (=100%) represent the mean ± S.D. (*n* = 3–4). Statistical analyses were performed using a one-way ANOVA with Dunnett’s test: *p* < 0.05 (* or §), *p* < 0.01 (** or §§), *p* < 0.001 (§§§); * different from control after 24 h, $ different from control after 72 h.

The influence of prenylflavonoids on the activity of DMEs was more pronounced in the differentiated cells. All prenylflavonoids caused a marked increase in GST and COMT activities (Figure 5a,c) after 72 h of incubation, ranging from 19.6% to 51.0%. In contrast, a prenylflavonoids-mediated inhibition of SULT activity, ranging from 19.3% to 32.7%, was observed (Figure 5b).

## 4. Discussion

Proliferating and differentiated CaCo-2 cells, one of the human colorectal cancer cell lines, were used in the present study. Proliferating CaCo-2 cells were chosen as a model of cancer cells, while differentiated CaCo-2 cells were selected as a model enterocyte-like cells, as this cell line upon differentiation loses its tumorigenic phenotype and displays characteristics of mature enterocytes (e.g., brush borders with microvilli) [26]. Moreover, several previous studies have reported an increase in the mRNA and protein expression of various enzymes during the differentiation of CaCo-2 cells [26,27,28,29], with GST, SULT and UGT ranking among the most highly upregulated genes [27]. In the study of Adnan et al. [30], the enzymatic activity, as well as protein expression of GSTA, was markedly increased with increasing the days of postconfluency. A proteomic analysis of CaCo-2 cells revealed a seven-fold, three-fold, six-fold and five-fold increase in the protein expression of GSTA1, GSTP, SULT1A3 and COMT, respectively. Moreover, the overall GST enzymatic activity was enhanced twice in differentiated cells [31]. All these data are in a good agreement with our obtained results (Table 1, Figure S1).

At the beginning, the cytotoxicity of prenylflavonoids was evaluated and compared among proliferating and differentiated cells. In this study, the highest cytotoxic effect towards proliferating, as well differentiated, CaCo-2 cells was observed for XH, which is in good agreement with our previously published results [8] as well as results obtained by other researchers using various cancer cells [32,33]. The cytotoxic activities of prenylflavonoids have been previously studied in various cancer cell lines and non-cancerous primary cell cultures [8,32,33,34,35]. The antiproliferative activity of natural prenylflavonoids seems to be highly cancer selective [32,35]. Moreover, prenylflavonoids show a higher anti-cancer activity than do their unprenylated counterparts, since the presence of the prenyl group may lead to an enhanced cellular uptake, reduced efflux and, thus, an increased intracellular activity of the compound [6,35]. In colorectal adenocarcinoma SW620 cells, XH cytotoxicity was accompanied by a deterioration in mitochondrial function as well as an increase in reactive oxygen species production [36]. The previously published low cytotoxicity of IXH and 8-PN in proliferating CaCo-2 cells corresponds well with our results [37].

One of the mechanisms of the chemopreventive activity of prenylflavonoids is the modulation of the mRNA and/or protein expression of detoxification enzymes (i.e., some DMEs and antioxidant enzymes). The GST and NAD(P)H:quinone oxidoreductase 1 (NQO1) rank among crucial detoxification enzymes that protect cells from oxidative damage, and they represent important targets for chemoprevention [14]. As mentioned above, the gene expression of detoxification enzymes is regulated via the modulation of the Keap1–Nrf2–ARE pathway [38] and modulation of this pathway can contribute to the chemopreventive activity of prenylflavonoids.

The second part of this project was focused on the possible modulatory effect of prenylflavonoids on the enzymatic activity and mRNA expression of GST, UGT, SULT and COMT. All dietary prenylflavonoids are metabolized by DMEs and thus they may compete with other substrates (i.e., other xenobiotics, drugs, as well as endogenous substances) of these enzymes in terms of binding to the active site of the enzyme. In rats and humans, oxidation, demethylation, hydration, glucuronidation and sulfonation reactions have been observed among the mechanisms of the formation of the metabolite [39,40]. In the stomach, a spontaneous conversion of XH to IXH takes place; IXH may be in turn converted to 8-PN by human hepatic cytochromes P450 (CYP) or gut microbial enzymes. The direct metabolic conversion of XH to desmethylxanthohumol, which is later converted into either 6-PN or into 8-PN, has also been described [10]. Therefore, the inhibition of CYP, UGT and SULT seems possible. A standardized hop extract, as well as isolated hop-derived prenylflavonoids, were shown to inhibit several human CYP isoforms, mainly CYP2C and CYP1A2 [3]. Moreover, prenylflavonoids may inhibit DMEs in a non-competitive or uncompetitive manner [11,12]. On the other hand, the ability of prenylflavonoids to induce the expression of DMEs via several signaling pathways has been demonstrated [4]. Due to the poor transition of prenylflavonoids to plasma, which may be caused by the specific binding of these compounds to the cytosolic proteins of enterocytes [41], the highest concentration of the prenylflavonoids is present in the intestinal cells, thus intestinal DMEs would be the most greatly affected by the modulatory effect of prenylflavonoids.

GSTA1 is highly expressed in many non-small-cell lung cancer cell lines and plays a crucial role in anti-cancer drug resistance. Its downregulation has been shown to lead to a suppression of tumor growth and the induction of cell apoptosis in the cancer cell line A549 [42]. Therefore, the downregulation of the GSTA1 mRNA expression caused by all tested prenylflavonoids in proliferating CaCo-2 cells observed in this study can be considered as positive regarding the suppression of cancer cell proliferation.

In non-cancerous cells and tissues, the upregulation of detoxification genes may be cytoprotective. In THLE-2 hepatocytes, a significant increase in the protein levels of GSTP and GSTT together with NQO1 and heme oxygenase upon XH treatment have been reported [15]. The administration of XH to human volunteers resulted in elevated plasmatic levels of GSTA, while levels of GSTP remained unchanged [43]. XH treatment also led to the diminished mRNA expression of liver SULT1A1 and UGT1A1 [16].

Some discrepancies among the enzymatic activities of DMEs and their mRNA expression levels have been observed mainly in the case of GST and COMT. The enzymatic activity of GST and COMT significantly increased, while the downregulation of the mRNA expression of GSTA1/2, GSTP and COMT was observed. A substantially low correlation between the enzymatic activity and mRNA expression of GSTA and NQO1 has been reported in primary rat hepatocytes treated with the prototypical aryl hydrocarbon receptor agonist β-naphthoflavone. On the other hand, a good correlation between those two parameters has been found in the case of CYP1A1/2 [44]. Taking a pharmacological/toxicological viewpoint into consideration, enzymatic activity is the most important indicator of a compound’s effect, as it ultimately affects the clearance of a metabolized drug [45].

An increase in the enzymatic activities of GST and COMT caused by prenylflavonoids, mainly 6-PN and 8-PN (Figure 5), can be considered as beneficial, since GST protects cells against potentially harmful electrophiles, and COMT prevents the conversion of hydroquinones and catechols into toxic quinones [46]. In rats, the administration of a hop extract significantly increased the enzymatic activity of NQO1 and GST in the liver [14]. In rats, after an acute ethanol administration, XH pretreatment protected various tissues against ethanol-induced oxidative stress in a dose-dependent manner via an increase in the activity of detoxification enzymes, including GST, reduced glutathione levels, and a reduction in reactive oxygen species levels [47]. In this study, all four of the tested prenylflavonoids significantly inhibited the SULT activity in differentiated CaCo-2 cells, while 6-PN and 8-PN caused a marked increase in the SULT activity in proliferating cells. Various flavonoids and isoflavonoids have been reported to be strong inhibitors of several SULT isoforms in vitro, e.g., SULT1A1 has been inhibited at submicromolar levels with luteolin, genistein and daidzein. A SULT inhibition by dietary compounds and environmental contaminants, the effects of which would be expected to be additive, could lead to the reduced sulfation of drugs (e.g., acetaminophen) and thus to altered pharmacological responses [48]. As the sulfation of 4-hydroxytamoxifen by SULT1A1 decreases therapeutic efficacy of tamoxifen in cancer cells [49], 6-PN and 8-PN, which increase the SULT activity in the proliferating cells, might reduce the tamoxifen treatment efficacy.

Differences in the effects of prenylflavonoids observed in proliferating and differentiated cells could be caused by a different transcriptome of those cell types. As is known, the expression of the drug-metabolizing enzymes, nuclear receptors and epigenetic regulators changes significantly during differentiation (or dedifferentiation in the case of cancer cells) [50,51].

## 5. Conclusions

The studied prenylflavonoids showed a higher cytotoxic activity towards proliferating cells than towards differentiated cells, with XH being the most active compound. The exposition of CaCo-2 cells to prenylflavonoids caused alterations in the activities and expression of phase II DMEs, although the modulatory effects of prenylflavonoids differed in proliferating cancer cells and in differentiated (enterocyte-like) cells. The prenylflavonoid-mediated increase of the GST and COMT activities together with the decrease of the SULT activities in enterocyte-like cells could indicate health benefits in terms of protection, but at the same time there is a possible risk of interactions between these compounds and concomitantly administered drugs, however, this hypothesis needs to be experimentally verified.

## Figures and Tables

**Table 1:** List of the primers used for RT-qPCR analysis of the selected genes.

Gene	Forward Primer	Reverse Primer
Target genes		
*COMT*	CACCATCGAGATCAACCCCG	TCATACTTCTTCTTCAGCTGGG
*GSTA1/2*	GTGCAGACCAGAGCCATTC	TCACCCAAATCTGCTATACCTTC
*GSTP1*	AGCCTTTTGAGACCCTGCTG	GTCAGCGAAGGAGATCTGGTC
*SULT1A1/2*	ATGGTTCAGCACACGTCGTT	GGACGGTGGTGTAGTTGGTC
*UGT1A6*	CCGGGGTCATGAGATTGTAGT	AGCTCTTCTTGGTCATACGGC
Reference genes		
*B2M*	TGCTGTCTCCATGTTTGATGTATC	TCTCTGCTCCCCACCTCTAAG
*GAPDH*	GAGTCCACTGGCGTCTTCAC	GAGGCATTGCTGATGATCTTGAG

**Table 2:** Specific activity and mRNA expression of phase II drug-metabolizing enzymes (DMEs) in proliferating and differentiated CaCo-2 cells.

Enzyme	Proliferating Cells	Differentiated Cells
GST		
Activity (nmol/min/mg)	163.9 ± 10.4	400.5 ± 33.8 ***
mRNA GSTA1/2	1.00 ± 0.16	1.46 ± 0.08 *
mRNA GSTP1	1.00 ± 0.36	1.33 ± 0.25
SULT		
Activity (pmol/min/mg)	86.6 ± 12.9	175.4 ± 52.3 *
mRNA SULT1A1/2	1.00 ± 0.23	2.44 ± 0.55 *
COMT		
Activity (nmol/min/mg)	3.1 ± 2.5	9.6 ± 1.2 **
mRNA COMT	1.00 ± 0.32	14.44 ± 4.83 **
UGT		
Activity	n.d.	n.d.
mRNA UGT1A6	n.d.	

Data are presented as the mean ± SD (*n* = 3). Statistical analyses were performed using a Student’s *t*-test: *p* < 0.05 (*), *p* < 0.01 (**), *p* < 0.001 (***); * different from proliferating cells after 24 h. GST: glutathione S-transferase; SULT: sulfotransferase; COMT: catechol-O-methyltransferase; UGT: UDP-glucuronosyltransferase; n.d.: not detected.

## Supplementary Materials

The following are available online at https://www.mdpi.com/2072-6643/12/7/2138/s1, Figure S1: The effect of prenylflavonoids (1 µM) on the normalized mRNA expression of GSTA1/2, GSTP1, SULT1A3, COMT and UGT1A6 in proliferating and differentiated CaCo-2 cells after 24 h and 72 h.
