# Supplementary material for: The Modulation of Phase II Drug-Metabolizing Enzymes in Proliferating and Differentiated CaCo-2 Cells by Hop-Derived Prenylflavonoids
Source: Nutrients. 2020 Jul 18;12(7):2138. doi: 10.3390/nu12072138 (PMC7400824; doi:10.3390/nu12072138)
Supplement: Supplementary file 1 [file nutrients-12-02138-s001.pdf]

Modulation of Phase II Drug-Metabolizing Enzymes in proliferating and differentiated CaCo-2 Cells by Hop-Derived Prenylflavonoids

Kateřina Lněničková [1], Michaela Šadibolová [2], Petra Matoušková [2], Barbora Szotáková [2], Lenka Skálová [2] and Iva Boušová [2,*]

[1] Faculty of Medicine and Dentistry, Palacký University, Hněvotínská 3, 775 15 Olomouc, Czech Republic; katerina.lnenickova@upol.cz (K.L.)
[2] Faculty of Pharmacy in Hradec Králové, Charles University, Heyrovského 1203, 500 05, Hradec Králové, Czech Republic; sadibolom@faf.cuni.cz (M.Š); matousp7@faf.cuni.cz (P.M.); szotakova@faf.cuni.cz (B.S.); skaloval@faf.cuni.cz (L.S.)
* Correspondence: Iva.Bousova@faf.cuni.cz (I.B.); Tel.: +420-495-067-406

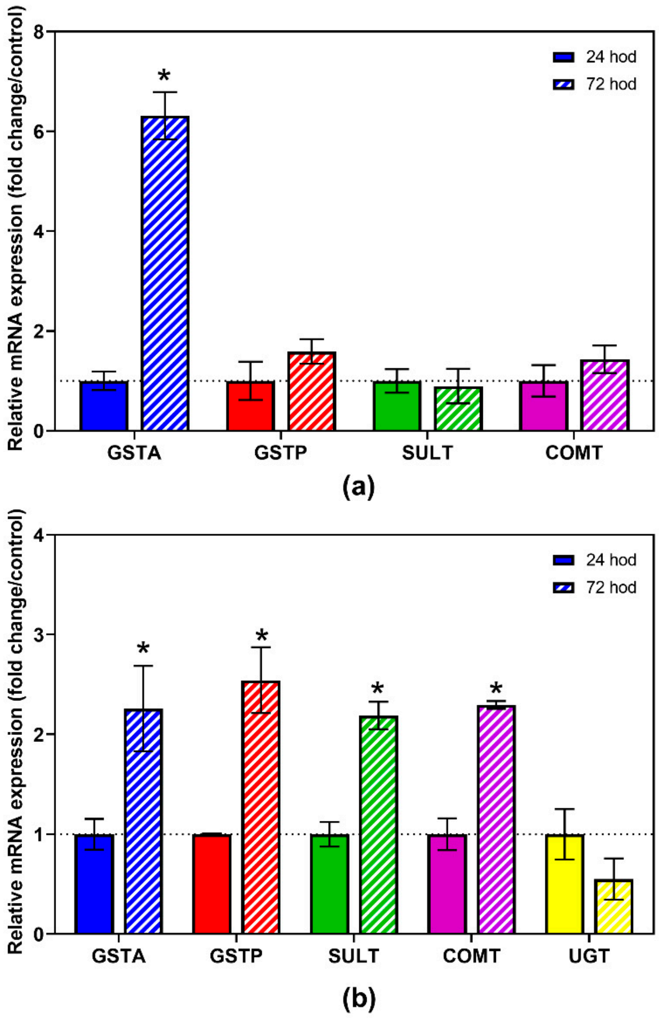

Figure S1. mRNA expression of phase II DMEs in proliferating (**a**) and differentiated (**b**) CaCo-2 cells after 24 h and 72 h. The normalized expression level was calculated using the $2^{-\Delta\Delta Ct}$ method with the geometric mean of GAPDH and B2M as a reference gene. Results are presented as the mean ± SD of 3 independent experiments. Statistical analyses were performed using Student's t-test with $p < 0.05$, * different from control after 24 h.
